# Screening for low bone mass with quantitative ultrasonography in a community without dual-energy X-ray absorptiometry: population-based survey

**DOI:** 10.1186/1471-2474-7-24

**Published:** 2006-03-09

**Authors:** Nan-Ping Yang, Ian Jen, Shao-Yuan Chuang, Shui-Hu Chen, Pesus Chou

**Affiliations:** 1 Community Medicine Research Center and Institute of Public Health, National Yang-Ming University, 155, Li-Nong Street, Section 2, Peitou, Taipei, ROC, Taiwan; 2 Department of Orthopedic Surgery, Tao-Yuan General Hospital, Department of Health, Executive Yuan, 1492, Jhong-Shan Road, Taoyuan, ROC, Taiwan; 3 Kin-Nin Country Health Center, 2, Fu-Hsing Road, Jinhu Town, Kinmen, ROC, Taiwan

## Abstract

**Background:**

Dual-energy x-ray absorptiometry (DXA) is the criterion standard to identify low bone mineral density (BMD), but access to axial DXA may be limited or cost prohibitive. We screened for low bone mass with quantitative ultrasonography (QUS) in a community without DXA, analyzed its reliability and obtained reference values and estimated the prevalence of low QUS values.

**Methods:**

We enrolled 6493 residents of Kinmen, Taiwan, and a reference group (96 men and 70 women aged 20–29 years) for this cross-sectional, community-based study. All participants completed a questionnaire and underwent ultrasonographic measurements. Reliability and validity of QUS measurements were evaluated. Broadband ultrasound attenuation (BUA) values were obtained and statistically analyzed by age, sex and weight. Annual loss of BUA was determined. Trends in the prevalence of QUS scores were evaluated.

**Results:**

Two QUS were used and had a correlation coefficient of 0.90 (p < 0.001). Calcaneal BUA was significantly correlated with BMD in the femoral neck (r = 0.67, p < 0.001) and BMD of the total lumbar spine (r = 0.59, p < 0.001). BUAs in the reference group were 92.72 ± 13.36 and 87.90 ± 10.68 dB/MHz for men and women, respectively. Estimated annual losses of calcaneal BUA were 0.83% per year for women, 0.27% per year for men, and 0.51% per year for the total population. The prevalence of severely low QUS values (T-score = -2.5) tended to increase with aging in both sexes (p < 0.001). Across age strata, moderately low QUS values (-2.5 < T-score < -1.0) were 31.6–41.0% in men and 23.7–38.1% in women; a significant trend with age was observed in men (p < 0.001).

**Conclusion:**

Age-related decreases in calcaneal ultrasonometry, which reflected the prevalence of low bone mass, were more obvious in women than in men.

## Background

Osteoporosis is an epidemiologic disorder that frequently results in fractures, psychological problems, social consequences, functional limitations and poor quality of life [1,2]. To reduce the incidence of osteoporotic fractures, individuals, especially postmenopausal women, who are at high risk for osteoporosis must be identified [3,4]. Dual-energy x-ray absorptiometry (DXA) is the most accurate clinical method to identify low bone mineral density (BMD) [4,5]. However, in some areas of the world, access to axial DXA is limited, or screening for low BMD with DXA is not cost effective [6]. Quantitative ultrasonography (QUS), which has been used to assess bone (especially calcaneal) status for almost 2 decades has proven to be widely and clinically useful [7-9].

**Figure 1:**
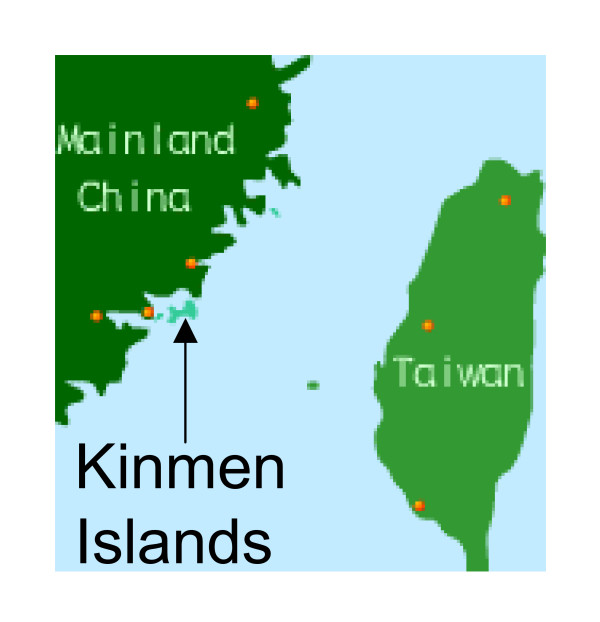
Geographic location of Kinmen between Taiwan and mainland China.

Kinmen, Taiwan, is a 176-km^2 ^island that is 154 miles west of Taiwan and 25 miles east of Mainland China. Two hospitals (one public and one military) and 41 physicians (as registered in 2000) serve the population. Because Kinmen is a military buffer district between Mainland China and Taiwan, Republic of China, (Figure 1), it has largely lacked medical resources. In Taiwan, more than 200 DXA machines available, but none are in Kinmen [10]. Although DXA is currently the criterion standard for diagnosing osteoporosis, referring high-risk individuals from Kinman for DXA is difficult because of travel costs. Therefore, inexpensive substitutes for DXA are needed. Bone QUS may be one such substitute.

The goals of our study were (1) to screen for low bone mass by using QUS in a community without DXA, (2) to conduct a reliability analysis and provide reference QUS values and (3) to estimate the prevalence of low QUS values.

## Methods

### Study design and screening

Between February 2000 and August 2003, a rural-community program in Kinmen was implemented and supervised by National Yang-Ming University, Taiwan; its institutional review board approved the study. This program was run in cooperation with the only public hospital and with the hygiene and medical department of the local government. Seven screening activities, with a duration of 10–14 days were performed within 42 months, A team including senior medical students of Yang-Ming University and local public health nurses performed most of the screening, always during the students' summer and winter vacations.

Kinmen has five townships with similar demographic compositions, and most people in Kinmen are involved in farming. We invited 7726 residents of these five areas aged 40 years or older at the time of the study to participate in this cross-sectional, community-based study. Military personnel were excluded.

After providing informed consent, all participants completed a self-administered questionnaire to provide baseline demographic data and underwent ultrasonographic measurements. Their body weight (in kilograms) and height (in centimeters) were measured, and their body mass index (in kilograms per square meters) was calculated. These data were used in the analysis of broadband ultrasound attenuation (BUA) values.

### Sonographic assessment

To ensure uniformity, BUA values were determined in each subject's right foot (rather than in his or her dominant foot). We used one of two gel-coupled QUS system (QUS-II calcaneal ultrasonometer; Metra Biosystems, Mountain View, CA, USA) approved by the US Food and Drug Administration. If the subject had a history of fracture or any bone disorder of the right foot, the left heel was evaluated. Several trained senior medical students performed the measurements in a temperature-controlled environment (room temperature about 23–27°C).

### Sonographic reliability analysis

For quality control and the evaluation of precision, the QUS devices were calibrated on a daily basis by using a phantom during the period of screening. The QUS devices were thought stable because no obvious instrumental shift was observed. The intra-test precision, as evaluated by using the coefficient of variation, was calculated from three repeated scans with repositioning in 25 volunteers in the first year of the study (ie, 2000). This short-term coefficient of variation in vivo (calculated as the root mean square) was 3.5% for BUA.

**Figure 2:**
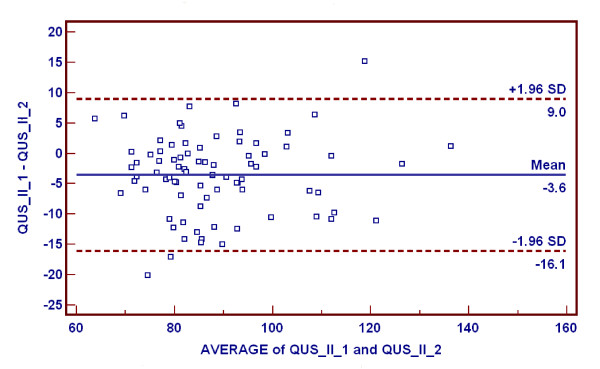
**Bland-Altman plot used to examine minor systematic differences between the two QUS machines**. Mean difference between the machines (QUS_II_1, QUS_II_2) was -3.56 ± 6.39 (standard deviation [SD]) dB/MHz.

Another group of 80 volunteers were examined by using the two QUS machines in the second year (ie, 2001). A graphical method, the Bland-Altman plot, was used to detect differences between the machines (Figure 2). The correlation coefficient was 0.90 (95% confidence interval 0.85–0.94, p < 0.001).

**Figure 3:**
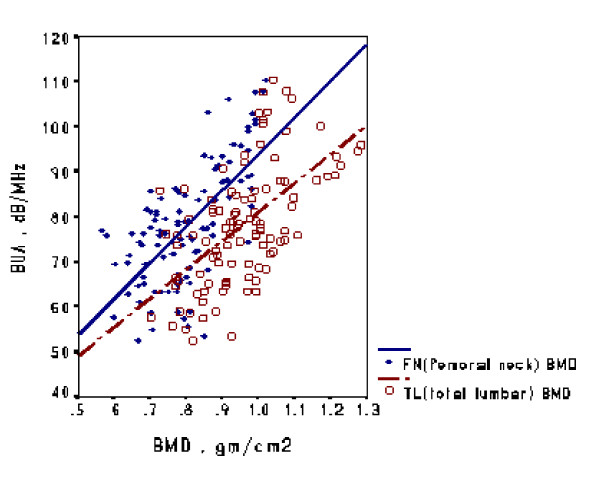
**Correlation of QUS and DXA measurements**. Mean (± standard deviation) BMD of the total lumbar (TL) region was higher than that of the femoral neck (FN) (0.96 ± 0.12 vs 0.80 ± 0.11; p < 0.001, paired t test).

To evaluate validity, QUS and DXA (QDR 4500; Hologic, Waltham, MA, USA) measurements were correlated during the same period in another group of 104 volunteers (26 men and 78 women; mean age ± standard deviation [SD], 46.2 ± 10.5 years). These subjects were scanned at a teaching hospital in Taiwan in the third year of the study (ie, 2002). The correlation observed between BMD measured in the femoral neck and BUA measured in the calcaneus (r = 0.67, p < 0.001) was significantly greater than that observed between BMD of the total lumbar spine and BUA in the calcaneus (r = 0.59, p < 0.001) (Figure 3).

### Preparation of sonographic reference data

In the second year (ie, 2001), 2% of the healthy population aged 20–29 years old in Kinmen were randomly selected to serve as a reference group. After excluding those with a fracture within 12 months, those with recent amenorrhea for more than 6 months and those with recent steroid use for more than 3 months, finally we enrolled 166 subjects (96 men and 70 women, sampling rates of 1.9% and 1.6% respectively; mean age ± SD, 23.2 ± 2.1 years). The BUAs were 92.72 ± 13.36 dB/MHz for the men and 87.90 ± 10.68 dB/MHz for the women. Therefore, the T-score for QUS for each subject was calculated as (BUA_individual _- BUA_reference_)/SD_reference_.

### Statistical analysis

Descriptive statistics included numbers and percentages of subjects and mean ± SD values. The Student t test and an analysis of variance were used to test for significant differences. Simple linear regression and Mantel-Haenszel chi-square tests were used to test for trends of the target variables when they were continuous and categorical scales, respectively. All statistical calculations were conducted by using software (SPSS for Windows 11.0 version; SPSS, Chicago, IL, USA). The Bland and Altman plot was drawn (Medcalc version 8.1.1).

Estimated annual losses (percentage per year) were calculated as the regression coefficient, or β value, for the variable age in multiple linear regression divided by the mean of BUA of the reference population. Multiple linear regression, with the dependent variable of BUA and the three independent variables of age, weight and BMI was performed by using a stepwise procedure. Finally, the regression models had three independent variables (age, weight and BMI; all p ≤ 0.01) for the total and male populations and two (age and weight; both p ≤ 0.01) in the female population.

## Results

### Baseline characteristics

Table 1 shows the demographic information for residents screened in 4 years. Clinicians in the screening program had invited 7726 residents. After they excluded subjects with a chronic systemic disorder (including chronic liver disease, chronic renal failure, chronic malabsorptive syndromes, gastrectomy, hyperparathyroidism, Cushing syndrome or long-term steroid use) and those with a fracture within 1 year, repeated visits, incomplete records or failed measurements from calcaneal QUS or anthropometry, 6493 participants were enrolled in our study. They included 2792 men (43.0%) and 3701 women (57.0%). Men weighed more than women in five age strata studied (p < 0.001), and BMIs were generally higher in women than in men (p < 0.001). Table 1 also shows the data for the reference subjects aged 20–29 years.

**Figure 4:**
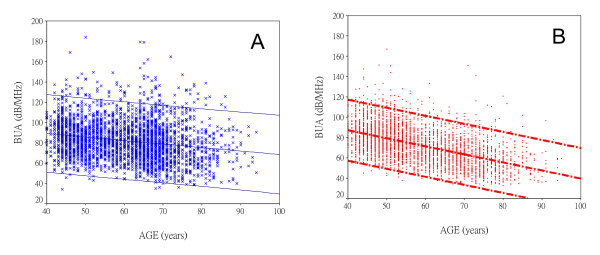
**Trends in BUA by sex**. Plots show individual BUA values and linear regression lines for the upper and lower limits of the 95% confidence interval in men (A) and women (B). The decline in BUA with age is more pronounced in women than in men (p < 0.001).

Figure 4 shows the relationship of BUA to age in both men and women. For both sexes, the decline of BUA with age was significant (p < 0.001), with a significant difference between men and women (p < 0.001).

### Trend of measured BUA values

Table 2 shows the BUA values of by sex and age. BUAs were higher in men than in women. In the total, male and female populations, BUA was negatively correlated with age (p < 0.001) but positively correlated with weight (p < 0.001) and positively correlated with BMI (p < 0.001).

Therefore, when we calculated the annual loss of BUA with increasing age, it had to be adjusted for weight and BMI by means of a multiple linear regression model. (The youngest group, aged 20–29 years, had the highest mean BUA, which was 90.69 dB/MHz). The regression coefficient, or β, for age in the multiple linear regression model was -0.246 for men, -0.756 for women, and -0.464 for the total population.

The estimated annual loss of calcaneal BUA for women was 0.83% per year, which was considerably more than the 0.27% per year for men and the 0.51% per year for the total population.

### Epidemiology of low bone mass, or low QUS values

Table 3 shows the three categorical distributions of bone mass, as represented by QUS measurements, by age and sex. When we applied the specific T-score designations based on the World Health Organization (WHO) criteria to the calcaneal QUS values, 36.5% of men had a T-score between -2.5 and -1.0, 11.7% had a T-score ≤ -2.5, whereas the percentages in women were 33.6% and 27.9%, respectively.

We observed significant trends for an increasing prevalence of severely low QUS values (T-score ≤ -2.5) with increasing age in both sexes. Prevalence of severely low QUS values were 3.9%, 7.1%, 13.5%, 21.4% and 22.9% in men aged 40–49, 50–59, 60–69, 70–79 and ≥80 years, respectively. These prevalence were marked increased in women, with rates of 6.7%, 16.5%, 41.0%, 58.6% and 70.4% in women aged 40–49, 50–59, 60–69, 70–79 and ≥80 years, respectively. Overall, the prevalence of moderately low QUS values (-2.5 < T-score < -1.0) ranged from 31.6% to 41.0% in men and from 23.7% to 38.1% in women, with a significantly increasing trend with age in men (p < 0.001).

### Comparison of prevalence and literature review

We reviewed the literature to understand the difference between our results and those of another similar survey of low bone mass. Most studies of QUS screening have focused on women, and the definition of low bone mass by using the T-scores based on QUS measurements are still controversial. Studies of women from neighboring Asian countries with the popular cutoff T-scores of -2.5 and -1.0 were suitable for comparison with ours.

Table 4 shows the prevalence of low QUS values among women from Japan [11], Korea [12], Taiwan [13], Vietnam [14] and Kinmen. The reference populations in Japan and Korea were the same age as ours (20–29 years). Our prevalence of severe low QUS values was higher than that in Japan for all age strata. However, moderately and severely low QUS values (T-scores < -1.0) were more prevalent in Japanese women than in the women in Kinmen for the age strata of 50–59, 60–69 and 70–79 years. The accumulated prevalence of severely low QUS values were 37.5% and 50.9% if the women in Kinmen aged ≥50 and ≥60 years, respectively. These rates were higher than those observed in Korea and Taiwan but lower than those in Vietnam. If the cutoff T-score was -1.0, the accumulated prevalence of low bone mass was still higher in Kinmen than in Korea or Taiwan. In general, the women in Kinmen had bone masses lower than those of women in neighboring Asian countries.

## Discussion

Because populations in Taiwan and elsewhere are aging, interest in the diagnosis, treatment and costs of osteoporosis has been renewed. In Taiwan, including Kinmen, where 22 million (mostly Chinese) people live, 8.6% of the population are elderly (aged 65 year or older), and their life expectancy is rapidly increasing [15]. In the elderly population, osteoporosis and related fractures are a major health problem. From the viewpoint of community medicine, population screening for osteoporosis in the elderly seems to increase their awareness of falling, and such screening is associated with a reduced fracture rate [16].

The first step of any action plan to prevent osteoporotic fractures is raising awareness [17]. This was why we investigated the use of QUS measurements and associated health education in Kinmen, where DXA is unavailable. Because of different geographic and environmental factors, the epidemiology of osteoporosis in Kinmen may differ from that in the West or in other Chinese areas. Therefore, subsequent epidemiologic analysis is needed.

Because QUS is safe, portable and inexpensive, it is a well-accepted instrument for screening for low BMD [18]. Several studies have shown significant correlations between calcaneal QUS values and calcaneus, hip, or spine BMD values measured with DXA [19-23]. A moderate and significant correlation was found, one also tested in our study; this can be applied as an alternative in populations other than those in the area we screened.

Although the diagnosis of osteoporosis by means of QUS remains controversial, the debate is due more to the limitations of present T-scores than to the technique [13,14,24-27]. Previous community-based studies of Japanese, Taiwanese and Vietnamese populations revealed that specific T-scores (same as the WHO criterion applied to calcaneal QUS) is reasonable when the reference group was selected from the same population as the group being examined [11,13,14].

By 1999, the US Food and Drug Administration had approved five QUS devices for use in the routine diagnosis of osteoporosis, for the determination of fracture risk, and for the monitoring bone changes [28]. The QUS-II machine (Metra Biosystems) was one of those devices. More than 20 ultrasound instruments are now widely used throughout the world. BUA results of the gel-coupled QUS-II ultrasound system (Metra Biosystems) are significantly associated with age, body weight, level of physical activity and dietary calcium intake [29]. One epidemiologic investigation performed with this ultrasonometer showed that age, body height and BMI are significant determinants of BUA for each sex, with the three factors collectively accounting for 25% of the total variance in BUA [14].

The general trend of age-related change in QUS variables was negative for both sexes, but it was more pronounced in women than in men. Linear regression analysis of BUA and age showed a decreased β value (regression coefficient) for women. In our study using QUS-II machines (Metra Biosystems), results were β = -0.76 (p < 0.001) for women and β = -0.25 (p < 0.001) for men on multiple regression. In Sweden [30], results were β = -0.39 (p < 0.001) for women and β = -0.24 (p < 0.01) for men on multiple regression. In Poland [31], results were β = -0.4 (p < 0.001) for women and β = -0.2 (p < 0.05) for men on multiple regression. Investigators in Sweden and for Poland used Achilles QUS systems. For the Chinese population of Taiwan [13], we previously used a UBIS 3000 machine. Our results were β = -0.5 (p < 0.001) for women and β = -0.1 (p < 0.001) on multiple regression. In Chinese women in Hong-Kong [32], the result obtained by using a Sahara QUS system were β = -0.44 (p < 0.001) on simple regression.

Compared with peak bone mass in the lumbar spine BMD, the annual bone loss in women was 3-fold that in men (1.28–1.60% vs 0.4–0.64%) after the age of 50 years [33]. These data were reported in a population-based, routine health study of healthy Asian individuals. We observed the same difference between men and women (0.27% vs 0.83% loss per year, respectively), but the absolute percentage of annual loss was approximately half of that in the aforementioned study. In a study of similar QUS screening in Rotterdam, the change in BUA units per year were -0.15% for men and -0.43% for women [34]; in a study in Norfolk, the rates were and -0.13% and -0.70%, respectively [35].

Previous studies of Taiwanese populations demonstrated that use of a specific T-score (the same as the WHO criterion applied to the calcaneal QUS value) is reasonable when the reference group is selected from the same population as the group being examined [13]. With similar methods, the prevalence of T-scores ≤ -2.5 calculated from a nationwide calcaneal QUS screening of bone mass of healthy Japanese women in the sixth, seventh, or eighth-and-older decades were 5.2%, 18.7% and 43.6%, respectively [11]; the prevalence of low QUS values (T-scores ≤ = 2.5) in Taiwanese women were 7.9, 21.7 and 34.5%, respectively [13]. Our rates of low QUS values in the sixth, seventh and eighth-and-older decades were 16.5%, 41.0% and 61.2% in women, and 7.1%, 13.5% and 21.7% in men.

In comparison with DXA-based BMD measurements at the hip, spine or forearm yield prevalence of osteoporosis of 14.8% in the sixth decade, 21.6% in the seventh decade, and 38.5% in the eight-and-older decade among Western women [36]. Prevalence of osteoporosis based on BMD measurements at the spine or hip are 24–38% in Japanese women older than 50 years [37] and 16–24% in Taiwanese women older than 80 years [38]. Our 37.5% rate of low bone mass (cutoff T-score = -2.5) in women from Kinmen older than 50 years was closest to the result from the Japanese Population-Based Osteoporosis Study [37].

QUS has great potential for widespread use owing to its portability, low cost, and lack of ionizing radiation. The usefulness of QUS in evaluating hip, spinal or other fractures in both women and men [39] has been proved and it is similar to that of BMD [40,41]. Cost-effectiveness analyses of QUS have shown a benefit in using ultrasonography as a selective population screening tool [42,43]. Therefore, QUS is worthy of evaluation in sex-, age- and diagnosis-specific populations to identify individuals with low bone mass, especially in the primary healthcare system. QUS measurement of the calcaneus may be an effective, acceptable, and useful tool for epidemiologic screening of low bone mass at a primary care units, especially those in areas where DXA is not available.

### Limitations

First, in 2000, the registered population of individuals aged 15 years or older in the four townships of Kinmen totaled 33,131. Because many of these individuals had been living in Taiwan because of their jobs, the true resident population was not investigated, and its response rate could not be calculated in the present study.

Second, because of the lack of normative data in Taiwanese populations for the DXA machine we used, the diagnostic power of QUS (including its sensitivity, specificity and positive predictive value) were lacking, and only the Pearson correlation between our DXA and QUS systems was evaluated.

Third, format criteria for QUS screening is not available up to now. Therefore, for an international comparison (ie. epidemiologic reviews in Asian women, showed in Table 4) of QUS surveys, different models (eg. age-range of the screened population, age-range of the reference population, the gender of targeted subjects, the instruments of QUS measurement et al) are not avoidable. To compare these studies, the same cutoff value of T-scores would be the only indicator.

## Conclusion

QUS was reliable in this community survey. Age-related decreases in calcaneal ultrasonometric values indicated that low bone mass was more obvious in women than men. The WHO criteria can reasonably be applied to calcaneal QUS when the reference group is selected from the same population as that being screened.

## List of abbreviations

BMD = bone mineral density

BMI = body mass index

BUA = broadband ultrasound attenuation

DXA = dual-energy x-ray absorptiometry

SD = standard deviation

WHO = World Health Organization

## Competing interests

The author(s) declare that they have no competing interests.

## Authors' contributions

The manuscript represents original work and the following authors have designed the study (NP Yang, Pesus Chou), gathered the data (NP Yang, SY Chuang, SH Cheng), analyzed the data (NP Yang, SY Chuang), wrote the initial drafts (NP Yang), and ensure the accuracy of the data and analysis (I Jen, Pesus Chou). All the authors participate in revisions of the manuscript and approve the final manuscript.

## Pre-publication history

The pre-publication history for this paper can be accessed here:



## Figures and Tables

**Table 1:** Demographic characteristics of study populations

				**Weight**		**BMI**	
					
**Age stratum (y)**	**Men**	**Women**	**Total**	Men*	Women*	Men*	Women*
Study group							
40–49	618 (34.8)	1156 (65.2)	1774 (27.3)	71.7 ± 10.3	59.3 ± 9.3^†^	24.9 ± 3.3	24.1 ± 2.6^†^
50–59	666 (40.2)	991 (59.8)	1657 (25.5)	70.1 ± 10.4	60.5 ± 9.4^†^	25.0 ± 3.2	25.0 ± 3.5^‡^
60–69	882 (52.7)	793 (47.3)	1675 (25.8)	65.7 ± 10.2	58.9 ± 9.6^†^	24.2 ± 3.2	25.3 ± 3.8^†^
70–79	482 (44.9)	592 (55.1)	1074 (16.5)	63.2 ± 9.9	57.2 ± 9.9^†^	23.8 ± 3.3	25.3 ± 4.0^†^
≥80	144 (46.0)	169 (54.0)	313 (4.8)	61.3 ± 10.6	54.0 ± 10.4^†^	23.6 ± 3.3	24.3 ± 4.0^‡^
Total	2792 (43.0)	3701 (57.0)	6493 (100)	67.4 ± 10.8	58.9 ± 9.7^†^	24.4 ± 3.3	24.8 ± 3.7^†^
Reference group							
20–29	96 (57.8)	70 (42.3)	166 (100)	62.4 ± 12.0	49.7 ± 4.8^†^	21.8 ± 2.7	19.3 ± 1.4^†^

Data are the number (percentage) or mean ± SD.

*p ≤ 0.01 on the analysis of variance for the study sub-group.

^†^p ≤ 0.01 on the t test between both gender.

^‡^Not significant.

**Table 2:** BUA (dB/MHz) by age and sex

**Age stratum (y)**	**Men**	**Women**
40–49	87.2 ± 18.0	82.8 ± 15.3
50–59	84.4 ± 18.4	77.2 ± 16.3
60–69	80.9 ± 20.4	66.0 ± 14.8
70–79	76.6 ± 20.9	59.9 ± 15.5
≥80	73.6 ± 19.1	56.2 ± 14.6

Data are the mean ± SD. On statistical analysis, p ≤ 0.001 for men versus women in all age strata, and p ≤ 0.001 for the trend on simple linear regression for men and women.

**Table 3:** Distribution of T-scores by age and sex

	**Normal T-Score**		**-2.5 < T-score < -1**		**T-score ≤ -2.5**	
			
**Age stratum (y)**	**Men**	**Women**	Men*	**Women^†^**	Men*	Women*
40–49	399 (64.6)	712 (61.6)	195 (31.6)	366 (31.7)	24 (3.9)	78 (6.7)
50–59	383 (57.5)	466 (47.0)	236 (35.4)	361 (36.4)	47 (7.1)	164 (16.5)
60–69	423 (48.0)	166 (19.4)	340 (38.5)	302 (38.1)	119 (13.5)	325 (41.0)
70–79	191 (39.6)	71 (12.0)	188 (39.0)	174 (29.4)	103 (21.4)	347 (58.6)
≥80	52 (36.1)	10 (5.9)	59 (41.0)	40 (23.7)	33 (22.9)	119 (70.4)
Total	1448 (51.9)	1425 (38.5)	1018 (36.5)	1243 (33.6)	326 (11.7)	1033 (27.9)

Data are the number (percentage).

*p ≤ 0.001 on Mantel-Haenszel chi-square test for the trend.

^†^Not significant on Mantel-Haenszel chi-square test for the trend.

**Table 4:** Prevalence of low bone mass (low QUS values), depended on different cutoff values of T-score, in Asian female populations

**Age stratum (y)**	**Present study**		Japan*		**Korea, Taiwan, and Vietnam**	
			
	**T-score -2.5**	**T-score -1.0**	**T-score -2.5**	**T-score -1.0**	**T-score -2.5**	**T-score -1.0**
40–49	6.7	38.4	0.7	32.1	ND	ND
50–59	16.5	52.5	5.2	64.8	ND	ND
60–69	41.0	79.1	18.7	88.7	ND	ND
70–79	58.6	88.0	43.6	95.2	ND	ND
≥50	37.5	72.0	ND	ND	11.8,^† ^20.0^‡^	26.8,^‡ ^56.0^§^
≥60	50.9	84.1	ND	ND	46.0,^† ^50.6^‡^	61.8^‡^

ND = no data.

*In Japan, 12,201 women aged 40 years or older were screened with Achilles, Lunar ultrasonometry at the calcaneus. Healthy subjects were aged 20–29 years [11].

^†^In South Korea, 552 women older than 50 years underwent Achilles, Lunar ultrasonometry at the calcaneus. Healthy subjects were aged 20–29 years [12].

^‡^In three Taiwanese communities, 2631 men and 3691 women aged 31 years or older were selected for UBIS-3000 ultrasonometry at the calcaneus. Healthy subjects were aged 31–40 years [13].

^§^In Vietnam, 668 men and 1390 women aged 18 years or older underwent QUS-II ultrasonometry at the calcaneus. Age of peak BUA was estimated to be 27 and 32 years in men and women, respectively [14].
